# A single-center culture-based study of *Helicobacter pylori* in Kazakhstan with regional meta-analysis of prevalence and antibiotic resistance

**DOI:** 10.3389/fmicb.2026.1747006

**Published:** 2026-01-22

**Authors:** Kaisar Dauyey, Gulnur Zhunussova, Jamilya Kaibullayeva, Yevgeniya Bondar, Arailym Yerzhan, Aliya Medetbekova, Aliya Kaisina, Alma Khabizhanova, Kanat Seitbekov, Yoshio Yamaoka

**Affiliations:** 1 Laboratory of Molecular Genetics, RSE "Institute of Genetics and Physiology" of the SC MSHE RK, Almaty, Kazakhstan; 2 Scientific-Research Institute of Cardiology and Internal Diseases, Almaty, Kazakhstan; 3 Department of Environmental and Preventive Medicine, Faculty of Medicine, Oita University, Yufu, Japan; 4 Research Center for Global and Local Infectious Diseases, Oita University, Yufu, Japan; 5 Department of Medicine, Gastroenterology and Hepatology Section, Baylor College of Medicine, Houston, TX, United States

**Keywords:** antimicrobial susceptibility test, *Helicobacter pylori*, Kazakhstan, meta-analysis, prevalence

## Abstract

**Background:**

*Helicobacter pylori* (*H. pylori*) is a major gastric pathogen and class I carcinogen that causes chronic gastritis, peptic ulcer, and gastric cancer if left untreated. However, evidence on *H. pylori* prevalence and antimicrobial resistance in Kazakhstan, a country with a high gastric cancer burden, remains scarce. This study presents the first culture-based epidemiological investigation of *H. pylori* at a single center in Almaty.

**Materials and methods:**

We conducted a cross-sectional study (2024–2025) of 150 dyspeptic patients in Almaty, Kazakhstan. A subset (*n* = 148) underwent rapid stool antigen (RAS) testing before gastric biopsy collection. Biopsy samples were cultured, and 86 (57.3%) yielded viable *H. pylori* isolates. Antimicrobial susceptibility testing by the agar dilution method was performed on these 86 isolates. Demographic and clinical data were analyzed, and a regional meta-analysis was conducted using data from recent studies across Central Asia and Russia to estimate pooled prevalence and clarithromycin resistance.

**Results:**

Among 148 patients tested by RAS, 137 were positive. Resistance rates among 86 isolates were 87.2% to metronidazole, 33.7% to clarithromycin, and 3.5% to amoxicillin; no resistance was detected to minocycline or sitafloxacin. Multidrug resistance (defined as resistance to two or more antibiotics) was observed in 34.8% of isolates. The pooled *H. pylori* prevalence across Central Asian studies was 70% (95% CI: 59–80%), and pooled clarithromycin resistance was 29% (95% CI: 10–53%).

**Conclusion:**

This study provides the first culture-based evidence of *H. pylori* infection and antimicrobial resistance in Kazakhstan. The high resistance to metronidazole and clarithromycin suggests a likely lower success of standard triple therapy in Almaty. Absence of resistance to minocycline and sitafloxacin supports their use in rescue regimens. These findings highlight the urgent need for national surveillance, updated treatment guidelines, and integration of molecular resistance monitoring to improve evidence-based management of *H. pylori* in Central Asia.

## 1 Introduction

The global burden of *Helicobacter pylori* (*H. pylori*) infection continues to rise in developing countries, with nearly two-thirds of all gastric cancer cases associated with *H. pylori* occurring in Asia (Park et al., 2025). Although the detailed mechanisms linking *H. pylori* infection to gastric carcinogenesis remain incompletely understood (Duan et al., 2025), approximately half of the world’s population carries the pathogen, predisposing them to chronic gastritis, peptic ulcer disease, and gastric adenocarcinoma (Wizenty and Sigal, 2025).

Despite extensive global research on *H. pylori* epidemiology, countries in Central Asia remain a geographical blind spot in global surveillance efforts. Kazakhstan, in particular, bears one of the highest gastric cancer burdens in the region (Chen et al., 2024). However, comprehensive studies that integrate field-based sampling, epidemiological analysis, and gold-standard culture-based methods, including antimicrobial susceptibility testing, are still lacking.

Antibiotic resistance in *H. pylori* has become a growing global concern, yet data from Central Asia remain limited and fragmented. Addressing this knowledge gap is essential for developing evidence-based treatment and eradication strategies tailored to the regional context. Therefore, the present study aims to fill this void by providing the first culture-based investigation of *H. pylori* infection and antimicrobial resistance in Kazakhstan.

East Asian countries such as Japan and Taiwan have established national surveillance systems and treatment guidelines for *H. pylori*, grounded in large-scale epidemiological studies that reflect trends in antibiotic resistance and population-based prevalence (Ono et al., 2025; Liang et al., 2020). These frameworks were initially built upon extensive strain culturing and phenotypic susceptibility testing. In Taiwan, national monitoring of antibiotic use has led to consistently low rates of amoxicillin resistance (<2–4%) and moderate rates of clarithromycin and metronidazole resistance (23.4 and 20.3%, respectively), reflecting long-standing regional prescribing patterns (Yeung and Lee, 2017). In contrast, studies from Japan and Korea report high rates of clarithromycin resistance (>15%) and an increasing prevalence of fluoroquinolone resistance, findings that have informed updates to national guidelines and encouraged the adoption of bismuth-based or susceptibility-guided regimens (Schulz et al., 2025).

High-quality, population-based studies on *H. pylori* prevalence and antibiotic resistance are crucial not only for pathogen eradication but also for the early detection and prevention of gastric cancer. Kazakhstan, despite its high gastric cancer burden, remains poorly characterized in this area, with no existing culture-based resistance profiling or population-level assessments. This study aims to fill this gap by using culture-based methods to determine infection prevalence and antibiotic resistance patterns among patients at a single center in Almaty, Kazakhstan. Our goal is to characterize *H. pylori* prevalence and resistance among dyspeptic patients using bacterial culture and to situate these findings within the context of available studies from other regions of Central Asia.

## 2 Materials and methods

### 2.1 Study design and sample collection

Between October 2024 and May 2025, we enrolled 150 adult patients presenting with dyspeptic symptoms at the Scientific-Research Institute of Cardiology and Internal Diseases in Almaty, Kazakhstan (Table 1). All participants provided written informed consent prior to inclusion and underwent upper gastrointestinal endoscopy.

**Table 1:** Study flow summary.

Stage	Number (Patients)
Enrolled and biopsied and cultured	150
RAS^*^ + Endoscopy report available	148
RAS positive	137
Culture-positive isolates and AST^+^	86

^*^RAS, rapid antigen stool testing.

^+^AST, Antibiotic susceptibility testing.

Eligible participants were adults aged ≥18 years with no prior history of *H. pylori* eradication therapy. To minimize confounding factors, all patients completed a structured questionnaire confirming that they had not used the following medications within 4 weeks before enrollment: H_2_ receptor antagonists, proton pump inhibitors (PPIs), bismuth compounds, antibiotics, nonsteroidal anti-inflammatory drugs (NSAIDs), or anticoagulants. Patients with a history of gastric surgery, significant comorbidities or malignancies, or those who were pregnant or breastfeeding were excluded from the study.

Rapid stool antigen testing (RAS) was performed using a commercially available immunochromatographic assay (Adtec Inc., Oita, Japan) according to the manufacturer’s instructions. The assay is an in-house tool issued by the manufacturer in collaboration with Oita University and is being evaluated in other clinical studies demonstrating high diagnostic accuracy (Passang Lhamo et al., 2025). In the present study, RAS was used solely as a supportive diagnostic tool and was not used to estimate infection prevalence or to guide antimicrobial resistance workflow, which were based solely on culture-positive biopsy samples.

During endoscopy, three gastric antral biopsy specimens were collected from each patient. Endoscopic findings were classified according to the Kimura–Takemoto system, which classifies the extent of gastric atrophy based on the anatomical location of the atrophy. Closed-type atrophy (C-1, C-2, C-3) indicates atrophy confined to the antrum or lesser curvature, while open-type atrophy (O-1, O-2, O-3) reflects continuous extension toward the gastric body and fundus. An increasing grade of mucosal atrophy corresponds to more pronounced mucosal atrophy. Two biopsy samples were immediately placed in in-house transport medium routinely used in Prof. Yamaoka’s laboratory for international *H. pylori* collaborative studies (Syam et al., 2015; Tourrette et al., 2024). At the same time, the third was preserved in RNAlater (Thermo Fisher Scientific, Waltham, MA, USA), kept on ice, and stored at −80 °C.

### 2.2 Sample transport and culture conditions

The biopsy samples were transported from the Institute of Genetics and Physiology in Almaty (Kazakhstan) to Oita University (Japan) in insulated containers with −30 °C ice packs, maintaining a cold chain throughout transport. International flight time, including door-to-door laboratory transaction, was approximately 24 h. Upon arrival, samples were stored at −80 °C before being thawed at 4 °C for immediate culture. Despite adherence to standard transport protocols, loss of bacterial viability during long-distance shipment is a known limitation of culture-based *H. pylori* studies and likely contributed to differences observed between RAS test and culture results.

*H. pylori* culture was performed using the homogenized biopsy specimen, which was inoculated onto *H. pylori-*selective media (Nissui Pharmaceutical Co., Ltd., Tokyo, Japan) and incubated for up to 10 days at 37 °C under microaerophilic conditions (10% O_2_, 5% CO_2_, and 85% N_2_). The obtained colonies were then sub-cultured onto Brucella agar (Becton Dickinson, Sparks, MD, USA) supplemented with 7% horse blood (Nippon Bio-test, Tokyo, Japan) without antibiotics. *H. pylori* colonies were determined based on the Gram staining results, bacterial morphology, and positive results for catalase, urease, and oxidase tests. These criteria are consistent with standard diagnostic protocols for *H. pylori*. Isolated and confirmed strains were preserved in Brucella broth (Becton Dickinson, Sparks, MD, USA) containing 10% glycerol and 10% horse serum and stored at −80 °C. Antimicrobial susceptibility testing was performed on culture-positive isolates (*n* = 86) using the agar dilution method. All cultures and antimicrobial susceptibility testing were performed in duplicate, and results were independently verified by two investigators. Reference strains routinely used in the laboratory were included for quality control as needed.

In this study, *H. pylori* culture was used as the reference standard for infection diagnosis, and RAS test results were evaluated in relation to culture results. Antibiotic resistance phenotypes were determined for all culture-confirmed isolates, and multidrug resistance (MDR) was defined as resistance to two or three antibiotics. Resistance pattern combinations were also summarized.

The European Committee on Antimicrobial Susceptibility Testing (EUCAST) clinical breakpoints (version 2022; https://www.eucast.org/clinical_breakpoints) were applied as follows: amoxicillin ≤ 0.125 mg/L, clarithromycin ≤ 0.5 mg/L, levofloxacin ≤ 1 mg/L, metronidazole ≤ 8 mg/L, and tetracycline ≤ 1 mg/L.

### 2.3 Statistical analysis and data visualization

All statistical analyses and data visualizations were performed using R software (version 4.3.1) (R Core Team, 2021). Summary tables were generated with the *gtsummary* and *gt* packages (Iannone et al., 2025; Sjoberg et al., 2021), and visual representations of antibiotic resistance and demographic variables were created using *ggplot2* (Wickham, 2016) to produce publication-ready figures. The exploratory nature of resistance profiling conducted in this epidemiological study did not require formal correction for multiple comparisons. The results for antibiotic susceptibility and MDR are interpreted descriptively.

To place our findings within a broader regional context, we conducted a secondary meta-analysis using data extracted from a recently published systematic literature review on *H. pylori* prevalence and antibiotic resistance in Central Asia. The final dataset included studies from Kazakhstan, Kyrgyzstan, Russia, and Uzbekistan published between 2000 and 2025 (Seisenbekova et al., 2025; Mežmale et al., 2021; Abylkasymova, 2012; Aldiyarova, 2011; Kulmambetova et al., 2015; Zhangabylov et al., 2002; Nurgalieva et al., 2002; Abdiev et al., 2008, 2010; Dzhumabaev et al., 2015; Dzhumabaev, 2008; Akbarova et al., 2017; Lazebnik et al., 2012; Karimov et al., 2019; Lavrinenko et al., 2025). Sample sizes and event counts for *H. pylori* prevalence and clarithromycin resistance were obtained directly from published tables and supplemented with data from the present study.

Pooled prevalence estimates were calculated using random-effects models with the Freeman–Tukey double arcsine transformation, as implemented in the *meta* and *metafor* R packages (Balduzzi et al., 2019; Viechtbauer, 2010). Statistical heterogeneity among studies was assessed using the *I*^2^ statistic, and forest plots were generated to visualize pooled estimates of *H. pylori* prevalence and clarithromycin resistance. The resulting pooled estimates were analyzed and interpreted cautiously, with emphasis on the overall picture of the region rather than on precise point estimates.

### 2.4 Ethics and regulatory compliance

The study protocol was approved by the relevant institutional ethics committees in Kazakhstan and Japan. Written informed consent was obtained from all participants prior to sample collection. International transfer of gastric biopsy specimens was conducted in compliance with institutional and national regulations governing the export and import of biological materials, including applicable ethics approvals and transfer agreements.

### 2.5 Study scope

This study was designed as a culture-based epidemiological approach to assess prevalence and perform phenotypic antimicrobial susceptibility testing of *H. pylori*. Although modern molecular and genomic methods are useful, they were beyond the scope of this work and are planned for future studies that will expand on this dataset.

## 3 Results

### 3.1 Baseline characteristics of the study population

A total of 150 dyspeptic patients provided gastric biopsy samples for analysis (Table 1). Among them, 148 (98.6%) underwent RAS testing; 137 (91.3%) were positive, indicating a high background prevalence of *H. pylori* infection among symptomatic patients. Viable *H. pylori* cultures were obtained from 86 of 150 biopsies (57%), and all isolates were subjected to antimicrobial susceptibility testing by the agar dilution method. The mean age of culture-positive patients was not significantly lower than that of culture-negative patients (42.9 ± 15.4 vs. 46.4 ± 15.6 years; *p* = 0.2). The sex distribution was comparable between groups (female 70% vs. 66%; *p* = 0.5), and two participants did not report their age (Table 2).

**Table 2:** Baseline characteristics by *H. pylori* culture result (*n* = 150) of patients who underwent endoscopic biopsy sampling.

Characteristic	*N* ^*^	Negative *N* = 64^1^	Positive *N* = 86^1^	*p*-value^2^
Age	148	46.4 ± 15.6	42.9 ± 15.4	0.2
Sex	150			0.5
Not reported		0 (0%)	2 (2.3%)	
Female		42 (66%)	60 (70%)	
Male		22 (34%)	24 (28%)	
RAS result	148			0.057
Negative		8 (13%)	3 (3.6%)	
Positive		56 (88%)	81 (96%)	
Endoscopy	150			0.020
No pathology		4 (6.3%)	0 (0%)	
Non-atrophic		24 (38%)	39 (45%)	
C-1		20 (31%)	17 (20%)	
C-2		7 (11%)	20 (23%)	
C-3		3 (4.7%)	6 (7.0%)	
O-1		4 (6.3%)	1 (1.2%)	
O-2		0 (0%)	1 (1.2%)	
O-3		1 (1.6%)	0 (0%)	
Not available		1 (1.6%)	2 (2.3%)	

1 Mean ± SD for continuous variables; *n* (%) for categorical variables.

2 Wilcoxon rank sum test; Pearson’s Chi-squared test.

Fisher’s exact test.

* *N* = 148 out of 150 of all cultured samples were obtained from patients who reported all clinical information, with two patients not reporting their age.

Kimura–Takemoto classification: C, closed-type atrophy; O, open-type atrophy; higher grades indicate more extensive gastric mucosal atrophy.

Endoscopic findings based on the Kimura–Takemoto system showed significant differences between groups (*p* = 0.02). Culture-positive patients demonstrated a higher proportion of C-2 and C-3 lesions (progressive gastric atrophy). Non-atrophic mucosa was slightly more common among culture-positive patients (45% vs. 38%). No patients in the culture-positive group had a normal endoscopic report, whereas 6.3% in the negative group did.

### 3.2 Antibiotic resistance and multidrug resistance patterns

Antimicrobial susceptibility testing was performed for 86 isolates (Table 3) against metronidazole, clarithromycin, amoxicillin, minocycline, and sitafloxacin. Metronidazole resistance was alarmingly high (75/86, 87.2%), confirming its limited utility in current empirical regimens. Clarithromycin resistance was found in 29 isolates (33.7%), while amoxicillin resistance remained rare (3/86, 3.5%). No resistance was detected to minocycline or sitafloxacin, suggesting that these agents may serve as viable options for second-line or rescue therapy.

**Table 3:** Antibiotic resistance and multidrug resistance profile among culture-positive *H. pylori* isolates (*n* = 86).

Antibiotic combination	No. resistant	% resistant
Overall antibiotic resistance in culture-positive *H. pylori* patients (*n* = 86)		
Amoxicillin	3/86	3.5
Clarithromycin	29/86	33.7
Metronidazole	75/86	87.2
Minocycline	0	0.0
Sitafloxacin	0	0.0
Pairwise antibiotic resistance combinations		
Clarithromycin + Metronidazole	27/86	31.4
Triple antibiotic resistance combinations		
Amoxicillin + Clarithromycin + Metronidazole	3/86	3.5

Multidrug resistance (MDR), defined as resistance to ≥ 2 antibiotics, was observed in 30 isolates (34.8%). Among them, 27 strains were dually resistant to clarithromycin and metronidazole, and 3 isolates were triple resistant to amoxicillin, clarithromycin, and metronidazole. These findings suggest the higher likelihood of emergence of complex resistance phenotypes in *H. pylori* circulating in the Almaty population.

### 3.3 Regional meta-analysis

**Figure 1:**
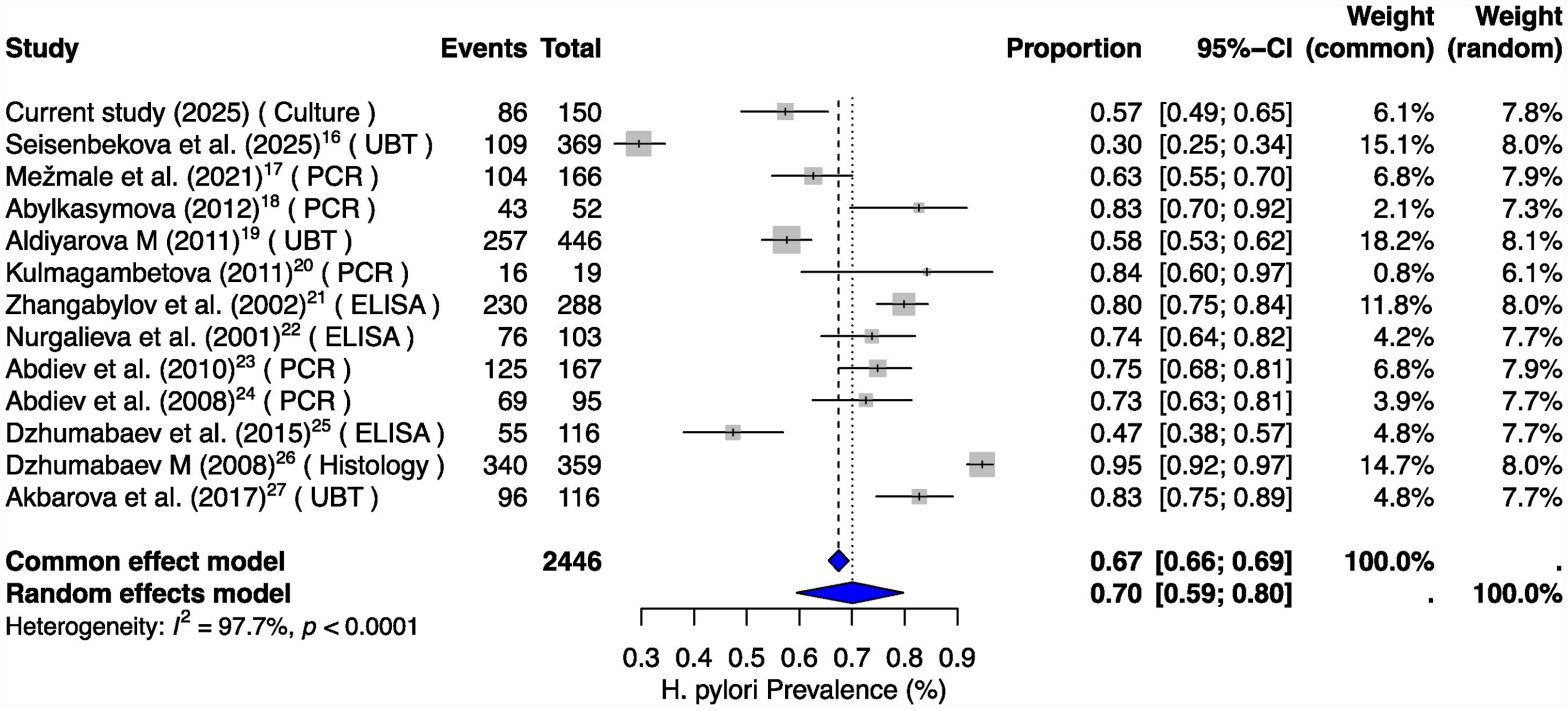
Forest plot of *Helicobacter pylori* prevalence across Central Asia. A random-effects meta-analysis was performed using published reports from Kazakhstan, Kyrgyzstan, and Uzbekistan, supplemented with our culture-based prevalence estimate. Our study identified *H. pylori* in 57% (86/150) of patients with evaluable biopsies and endoscopy. Pooled prevalence across Central Asia was 70% (95% CI: 58–80%; *I*^2^ = 97.7%), reflecting both high endemicity and methodological heterogeneity among studies. Events are defined as positive *H. pylori* infection cases relative to the total number of patients recruited in each study.

To contextualize our results, we performed a secondary meta-analysis of eligible studies from Kazakhstan, Kyrgyzstan, and Uzbekistan. The pooled prevalence of *H. pylori* infection was 70% (95% CI: 59–80%; *I*^2^ = 97.7%) (Figure 1), consistent with high endemicity across Central Asia. Our culture-based prevalence in Almaty (57.3%) closely aligned with previous reports from urban Kazakhstan, supporting regional heterogeneity ranging from 30 to 85%.

**Figure 2:**
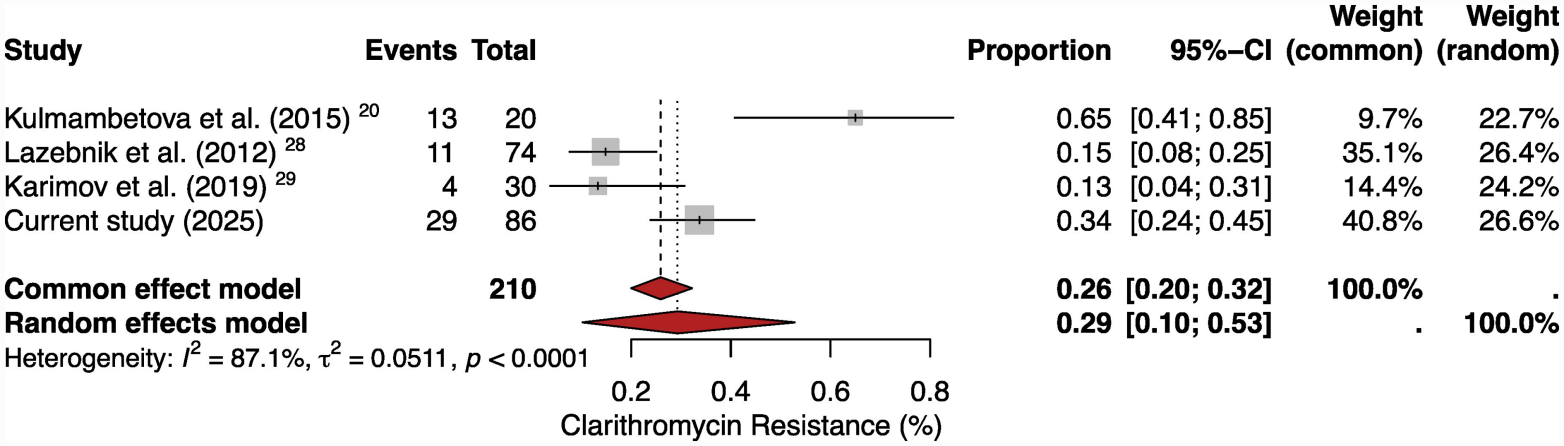
Forest plot of clarithromycin resistance among *H. pylori* isolates in Central Asia and Russia. Pooled clarithromycin resistance was estimated from regional studies using phenotypic or molecular methods. Our study contributed culture-based data from 86 isolates, with a resistance rate of 33.7% (29/86). The pooled resistance across was 29% (95% CI: 10–53%; *I*^2^ = 87.1%), underscoring the growing challenge of clarithromycin resistance in the region. Events are defined as clarithromycin-resistant *H. pylori* cases or strains relative to the total number of patients recruited in each study.

Regarding antimicrobial resistance, four prior reports yielded a pooled clarithromycin resistance rate of 29% (95% CI: 10–53%; *I*^2^ = 87.1%) (Figure 2). The resistance level observed in our study (33.7%) corresponds well with this regional estimate, validating the reproducibility of our findings. Collectively, these data highlight both the high burden and growing complexity of *H. pylori* infection and antibiotic resistance patterns in Central Asia.

## 4 Discussion

Our study provides the first culture-based epidemiological and antimicrobial resistance data for *H. pylori* in Kazakhstan, complemented by a regional meta-analysis. Three main findings emerge. First, the burden of *H. pylori* infection in Almaty remains high, with more than half of symptomatic patients harboring viable bacteria. Second, *in vitro* antimicrobial resistance patterns observed for metronidazole (87.2%) and clarithromycin (33.7%) suggest a reduced likelihood of treatment success with the standard triple therapy; however, this cannot be reliably assessed without measured clinical eradication rates. Third, our pooled regional estimates of 70% prevalence and 29% clarithromycin resistance position Kazakhstan within the broader Central Asian context, demonstrating both consistency with neighboring countries and unique resistance features (Karimov et al., 2019; Lavrinenko et al., 2025).

RAS detected *H. pylori* antigens in a high proportion of patients, including some with culture-negative biopsies, suggesting detection of non-viable bacteria. Among culture-positive patients, 96% were RAS-positive, whereas 88% of culture-negative patients were also RAS-positive (*p* = 0.057). This near-significant difference supports the reliability of RAS as a practical screening tool in resource-limited clinical settings.

### 4.1 Comparison with previous studies in Central Asia

Our findings fill an essential gap in the existing literature for this region. The most recent systematic review on Central Asia reported *H. pylori* prevalence in Kazakhstan ranging from 62.4 to 85.3%, compared to Uzbekistan (72–80%) and Kyrgyzstan (51–81%) (Seisenbekova et al., 2025). However, variations in diagnostic methods may have affected these outcomes. In Kazakhstan, the most frequently reported method has been the enzyme-linked immunosorbent assay (ELISA), whereas clinicians in Kyrgyzstan and Uzbekistan commonly rely on polymerase chain reaction (PCR), histological confirmation, or urea breath tests (UBT) (Lavrinenko et al., 2025).

### 4.2 Within-country variability and risk factors

Within Kazakhstan, regional estimates vary considerably. A recent hospital-based study in Karaganda reported an *H. pylori* prevalence of only 27.6% (Seisenbekova et al., 2025). This wide variation underscores the need for a nationwide study using standardized diagnostic methods to accurately quantify the actual burden of active infection. Previous research has identified age and dietary factors, particularly high salt intake, as significant risk factors for *H. pylori* infection in Kazakhstan (Mežmale et al., 2021). In our study, there was no association between age and culture positivity, suggesting that active infection may be equally prevalent among younger and older patients. Because most earlier studies in Kazakhstan relied on antibody-based ELISA with modest sensitivity and specificity, the distinction between past and active infection has been unclear. Culture-based approaches, though technically demanding, remain essential for accurate diagnosis and antibiotic susceptibility testing, which are rarely implemented in routine clinical practice across Central Asia.

### 4.3 Clinical and microbiological implications

The antimicrobial susceptibility results raise significant concerns about the efficacy of currently recommended *H. pylori* eradication regimens. High resistance rates to metronidazole and clarithromycin suggest a diminished likelihood of success of standard triple therapy. Recent global consensus data indicate that clarithromycin resistance exceeding 15% and metronidazole resistance exceeding 40% are each associated with a lower eradication success rate in patients receiving standard triple therapy (Liou et al., 2025). Although amoxicillin resistance was rare (3.5%), the few resistant strains were concurrently resistant to clarithromycin and metronidazole, indicating the emergence of multidrug-resistant (MDR) *H. pylori* phenotypes. The absence of resistance to sitafloxacin and minocycline suggests that these agents may serve as viable components of second-line or rescue therapy regimens. Continuous monitoring of resistance patterns is essential to guide empirical therapy, as seen in Japan and Taiwan (Ono et al., 2025; Liou et al., 2025), where phenotype-guided regimens have improved eradication success rates. These inter-country differences we have observed in Kazakhstan also reflect disparities in healthcare access, diagnostic capacity, and antibiotic prescription practices. Empirical antibiotic use remains relatively common in Kazakhstan, which may have contributed to the higher resistance rates observed compared with its neighbors. While PCR and ELISA are widely available and helpful for non-invasive diagnosis, the lack of culture-based and genotypic testing limits the ability to monitor antibiotic resistance or virulence markers. There is a pressing need for laboratories in Central Asia to adopt such gold-standard approaches, which are routinely used in international *H. pylori* eradication guidelines (Bui et al., 2025; Yamaoka, 2024).

### 4.4 Regional genetic context

Despite the high prevalence of *H. pylori* infection, genetic data on antibiotic resistance from Central Asia remain scarce. A previous study in central Kazakhstan (*n* = 20) identified clarithromycin-associated 23S rRNA mutations in 13 strains, *rdxA* mutations in 8 strains, and *PBP1a* mutations associated with amoxicillin resistance in 1 strain (Kulmambetova et al., 2015). In Uzbekistan, 23S rRNA A2142G/C mutations were found in 4 of 30 (13.3%) isolates (Karimov et al., 2019), whereas in Kyrgyzstan, resistance rates to clarithromycin and metronidazole were approximately 16.2 and 45%, respectively (Moldobaeva et al., 2014). Compared with these reports, our study demonstrates a higher phenotypic resistance to metronidazole, moderately elevated clarithromycin resistance, and clear evidence of emerging MDR *H. pylori* in Kazakhstan.

### 4.5 Public health implications

Given Kazakhstan’s high gastric cancer burden, with 27,467 new cases reported between 2009 and 2018 (Taszhanov et al., 2022), our results emphasize the urgent need for coordinated national action. A combined approach integrating non-invasive screening, culture-based diagnosis, and molecular surveillance will be essential to develop evidence-based treatment guidelines tailored to regional resistance profiles. The current study, though limited to the Almaty region, represents the first step toward establishing a national *H. pylori* surveillance network. Expanding this framework to the other areas will provide comprehensive data to inform clinical protocols not only in Kazakhstan but also in neighboring Central Asian countries.

### 4.6 Limitations and technical considerations

Several limitations should be acknowledged. This study was conducted at a single center in Almaty among symptomatic patients referred for endoscopy; therefore, the findings may not be generalizable to the entire country without corroborating evidence from other regions or population-based surveys. The relatively small sample size of positive bacterial cultures obtained (*n* = 86) was insufficient for multivariable regression analyses examining sociodemographic factors associated with the spread of *H. pylori* in the region. Additionally, the identification of MDR in 34.8% of isolates, coupled with the small number of triple-resistant strains, does not allow a clear interpretation of antibacterial resistance patterns or conclusions regarding clinical outcomes in the studied population. Nevertheless, the occurrence of clarithromycin–metronidazole double resistance is clinically alarming and warrants in-depth investigation. In the future, multi-center, population-based studies will be required to identify independent predictors of *H. pylori* prevalence and MDR in Kazakhstan and Central Asia.

Among the 148 patients who underwent RAS testing, 81 of 86 culture-positive patients (96%) were also RAS-positive, while 56 of 64 culture-negative patients (88%) were RAS-positive. The partial mismatch between RAS and culture results may stem from methodological constraints: RAS detects *H. pylori* antigens, whereas culture requires viable bacteria. Because biopsy samples were transported from Kazakhstan to Japan for culturing, bacterial cell death during shipment may have led to culture-negative yet RAS-positive results. The higher proportion of RAS-positive results among culture-negative patients (88%) indicates that culture likely underestimates infection in the presence of non-viable or coccoid forms of the bacteria, particularly under conditions requiring international sample transport.

The very high heterogeneity observed in the meta-analysis (*I*^2^ > 97% for prevalence and *I*^2^ > 85% for clarithromycin resistance) reflects methodological differences across studies, including diagnostic approaches (ELISA, PCR, UBT, histology and culture), population characteristics, and geographic regions. Therefore, pooled estimates should be interpreted descriptively for assessing the regional burden rather than used to make precise evaluations for policy-making and clinical protocols.

In future investigations, establishing in-country culture and susceptibility testing facilities will be essential to reduce sample degradation and achieve more accurate estimates of infection rates and resistance profiles. Our findings also indicate that RAS is highly sensitive but has limited practical value in the early stages of epidemiological work within a single-center design. While culture may underestimate infection under technically limited conditions, future surveillance programs should incorporate both culture-based and noninvasive diagnostic methods to ensure comprehensive detection of *H. pylori* infection in multicenter studies across Kazakhstan and Central Asia.

### 4.7 Future directions and conclusion

Considering these findings and limitations, we present the need for a coordinated national survey on *H. pylori* infection and antibiotic resistance in Kazakhstan. The high rates of resistance to clarithromycin (33.7%) and metronidazole (87.2%) observed indicate that current first-line therapies may be less effective than expected in a subset of patients. Based on our results, we propose the following clinical considerations for Almaty: (i) restrict clarithromycin-based triple therapy if other population-based studies confirm the resistance exceeding 15%; (ii) prioritize bismuth-based quadruple therapy in cases of confirmed clarithromycin-metronidazole MDR; (iii) reserve sitafloxacin or minocycline for rescue therapy; and (iv) establish local culture and molecular resistance microbiological facilities for ongoing monitoring and surveillance of antibiotic resistance. Future work should extend surveillance to other regions and incorporate molecular and genomic analyses to track geographic variation and resistance patterns.

Kazakhstan, as one of the countries with the highest gastric cancer incidence in Central Asia, must prioritize *H. pylori* control as part of its national cancer prevention strategy. This includes noninvasive population screening, the establishment of centralized diagnostic capacity, and the revision of treatment guidelines. Our study provides the first culture-based evidence supporting these initiatives, thereby bridging a critical research gap in understanding the regional patterns of *H. pylori* prevalence and antimicrobial resistance.

## Data Availability

The raw data supporting the conclusions of this article will be made available by the authors, without undue reservation.

## References

[ref1] Abdiev S. Ahn K. S. Khadjibaev A. Malikov Y. Bahramov S. Rakhimov B. . (2010). *Helicobacter pylori* infection and cytokine gene polymorphisms in Uzbeks. Nagoya J. Med. Sci. 72, 167–172. 20942272 PMC11259146

[ref2] Abdiev S. Ahn K. S. Rahimov B. Bahramov S. Malikov Y. R. Khadjibaev A. M. . (2008). *Helicobacter pylori* infection and peptic ulcer disease in Uzbekistan. Helicobacter 13, 304–305. doi: 10.1111/j.1523-5378.2008.00613.x, 18665941

[ref3] Abylkasymova К. (2012). Comparative analysis of the sensitivity of laboratory diagnostic methods (ELISA and PCR) for *Helicobacter pylori* infection (in Russian). Bull Kazn 2:271.

[ref4] Akbarova G. M. Moldobayeva M. S. Ulamadylova А. У. (2017). Risk factors for *Helicobacter Pylori* infection in indigenous people in the regions of Kyrgyzstan (in Russian). Bull KSMA Named IK Akhunbaeva 1, 47–51.Torokul kyzy, E.

[ref5] Aldiyarova M. A. (2011). Prevalence and features of infection caused by *Helicobacter pylori* in the indigenous population of the southern region of Kazakhstan. Epidemiol Infect Dis 1, 25–27.

[ref6] Balduzzi S. Rücker G. Schwarzer G. (2019). How to perform a meta-analysis with R: a practical tutorial. Evid. Based Ment. Health 22, 153–160. doi: 10.1136/ebmental-2019-300117, 31563865 PMC10231495

[ref7] Bui P. H. Cao T. N. M. Tran T. T. Matsumoto T. Akada J. Yamaoka Y. (2025). Identification of genetic determinants of antibiotic resistance in *Helicobacter pylori* isolates in Vietnam by high-throughput sequencing. BMC Microbiol. 25:264. doi: 10.1186/s12866-025-03990-w, 40312324 PMC12046797

[ref8] Chen Y. C. Malfertheiner P. Yu H. T. Kuo C. L. Chang Y. Y. Meng F. T. . (2024). Global prevalence of *Helicobacter pylori* infection and incidence of gastric cancer between 1980 and 2022. Gastroenterology 166, 605–619. doi: 10.1053/j.gastro.2023.12.022, 38176660

[ref9] Duan Y. Xu Y. Dou Y. Xu D. (2025). *Helicobacter pylori* and gastric cancer: mechanisms and new perspectives. J. Hematol. Oncol. 18:10. doi: 10.1186/s13045-024-01654-2, 39849657 PMC11756206

[ref10] Dzhumabaev M. N. (2008). Regional and ethnic features of prevalence *Н. рylori* associated diseases (in Russian). Izv Vysshih Uchebnyh Zaved Kyrg IVK 6, 236–238.

[ref11] Dzhumabaev M. N. Dzhumanova R. G. Sabirov I. S. (2015). The interdependence between smoking, alcohol, tooth pathology and prevalence of *Helicobacter Pylori* among ethnic groups in Kyrgyzstan (in Russian). Eksp Klin Gastroenterol Exp Clin Gastroenterol 6, 16–20. 26817100

[ref12] Iannone R. Cheng J. Schloerke B. Hughes E. Lauer A. Seo J. . (2025). Gt: Easily create presentation-ready display tables. Vienna: The R Foundation.

[ref13] Karimov M. M. Sobirova G. N. Saatov Z. Z. Islamova S. Z. Rustamova S. T. (2019). Prevalence and molecular-genetic characteristics of Helicobacter pylory in Uzbekistan. Eff. Pharmacother. 15, 48–51. doi: 10.33978/2307-3586-2019-15-28-48-51

[ref14] Kulmambetova G. N. Bekenova E. E. Tujakova A. K. Kozhakhmetov S. S. Logvinenko A. A. Sukashev A. T. . (2015). Antibiotic resistance of *Helicobacter pylori* isolates from Kazakh patients. Biotechnol. Theory Pract. 4, 4–9.

[ref15] Lavrinenko A. Seisenbekova A. Turemuratova A. Shkreba A. Yukhnevich Y. (2025). Review of methods for detection *Helicobacter pylori* in Kazakhstan. JGH Open 9:e70101. doi: 10.1002/jgh3.70101 39830988 PMC11740085

[ref16] Lazebnik L. Belousova N. Bordin D. Mikheyeva O. Dubtsuova Y. Vorobyeva N. . (2012). *Helicobacter pylori* resistance to clarithromycin in Moscow and propolis as a means increasing eradication effectiveness. Exp. Clin. Gastroenterol. 8, 10–14.

[ref17] Liang C. M. Tai W. C. Hsu P. I. Wu D. C. Kuo C. H. Tsay F. W. . (2020). Trend of changes in antibiotic resistance in *Helicobacter pylori* from 2013 to 2019: a multicentre report from Taiwan. Ther. Adv. Gastroenterol. 13:1756284820976990. doi: 10.1177/1756284820976990, 33354229 PMC7734532

[ref18] Liou J. M. Malfertheiner P. Hong T. C. Cheng H. C. Sugano K. Shah S. . (2025). Screening and eradication of *Helicobacter pylori f* or gastric cancer prevention: Taipei global consensus II. Gut 74, 1767–1791. doi: 10.1136/gutjnl-2025-336027 40912906

[ref19] Mežmale L. Polaka I. Rudzite D. Vangravs R. Kikuste I. Parshutin S. . (2021). Prevalence and potential risk factors of *Helicobacter pylori* infection among asymptomatic individuals in Kazakhstan. Asian Pac. J. Cancer Prev. 22, 597–602. doi: 10.31557/APJCP.2021.22.2.597, 33639679 PMC8190350

[ref20] Moldobaeva M. S. Elistratov A. A. Tolombaeva A. A. (2014). Modern approaches to the cancer prevention in HP-associated gastritis (in Russian). Her KRSU 14, 102–106.

[ref21] Nurgalieva Z. Z. Malaty H. M. Graham D. Y. Almuchambetova R. Machmudova A. Kapsultanova D. . (2002). *Helicobacter pylori* infection in Kazakhstan: effect of water source and household hygiene. Am. J. Trop. Med. Hyg. 67, 201–206. doi: 10.4269/ajtmh.2002.67.201, 12389948

[ref22] Ono A. Tanaka S. Sawada N. Goto A. Tsugane S. Muraki I. . (2025). *Helicobacter pylori* eradication and gastric cancer prevention in a pooled analysis of large-scale cohort studies in Japan. Sci. Rep. 15:21307. doi: 10.1038/s41598-025-00713-z, 40593877 PMC12215362

[ref23] Park J. Y. Georges D. Alberts C. J. Bray F. Clifford G. Baussano I. (2025). Global lifetime estimates of expected and preventable gastric cancers across 185 countries. Nat. Med., 31, 3020–3027. 40624406 10.1038/s41591-025-03793-6 PMC12443596

[ref24] Passang Lhamo S. Matsumoto T. Tshering K. Pradhan B. Akada J. Yamaoka Y. (2025). Prevalence of and risk factors for *Helicobacter pylori* infection in children under 64 months in Thimphu, Bhutan, and introducing the new in-house immunochromatography test kit: a cross-sectional study. Gut Pathog. 17:39. doi: 10.1186/s13099-025-00715-2 40468433 PMC12139328

[ref25] R Core Team (2021). R: a language and environment for statistical computing. Vienna, Austria: R Foundation for Statistical Computing.

[ref26] Schulz C. Liou J. M. Alboraie M. Bornschein J. Campos Nunez C. Coelho L. G. . (2025). *Helicobacter pylori* antibiotic resistance: a global challenge in search of solutions. Gut:gutjnl-2025-335523. doi: 10.1136/gutjnl-2025-335523 40645767

[ref27] Seisenbekova A. Laryushina Y. Yukhnevich Y. Lavrinenko A. Shkreba A. (2025). Prevalence and risk factors of *H. pylori* infection among outpatient in Karaganda city (Kazakhstan). Future Sci. OA 11:2461429. doi: 10.1080/20565623.2025.2461429, 39927633 PMC11812317

[ref28] Seisenbekova A. Lavrinenko A. Laryushina Y. Otemis A. Volnovaya O. Solomadin M. . (2025). Antibiotic resistance of *Helicobacter pylori*: data from Central Asia. J. Clin. Med. Kaz. 22:62. doi: 10.1080/20565623.2025.2461429

[ref29] Sjoberg D. D. Whiting K. Curry M. Lavery J. A. Larmarange J. (2021). Reproducible summary tables with the gtsummary package. R J. 13, 570–580. doi: 10.32614/rj-2021-053

[ref30] Syam A. F. Miftahussurur M. Makmun D. Nusi I. A. Zain L. H. Zulkhairi A. F. . (2015). Risk factors and prevalence of *Helicobacter pylori* in five largest islands of Indonesia: a preliminary study. PLoS One 10:e0140186. doi: 10.1371/journal.pone.0140186 26599790 PMC4658100

[ref31] Taszhanov R. Telmanova Z. Zhadykova Y. Akhmetova L. Zhantureyeva A. Bukeyeva Z. . (2022). Geographic variability of gastric cancer incidence in Kazakhstan. Asian Pac. J. Cancer Prev. 23, 1935–1944. doi: 10.31557/apjcp.2022.23.6.1935, 35763634 PMC9587815

[ref32] Tourrette E. Torres R. C. Svensson S. L. Matsumoto T. Miftahussurur M. Fauzia K. A. . (2024). An ancient ecospecies of *Helicobacter pylori*. Nature 635, 178–185. doi: 10.1038/s41586-024-07991-z, 39415013 PMC11541087

[ref33] Viechtbauer W. (2010). Conducting meta-analyses in R with the metafor package. J. Stat. Softw. 36, 1–48. doi: 10.18637/jss.v036.i03

[ref34] Wickham H. (2016). ggplot2: elegant graphics for data analysis. New York: Springer-Verlag.

[ref35] Wizenty J. Sigal M. (2025). *Helicobacter pylori*, microbiota and gastric cancer—principles of microorganism-driven carcinogenesis. Nat. Rev. Gastroenterol. Hepatol. 22, 296–313. doi: 10.1038/s41575-025-01042-2, 40011753

[ref36] Yamaoka Y. (2024). Revolution of *Helicobacter pylori* treatment. J. Gastroenterol. Hepatol. 39, 1016–1026. doi: 10.1111/jgh.16526, 38414319

[ref37] Yeung C. Y. Lee H. C. (2017). Paediatric *Helicobacter pylori* infection in Taiwan: current status and perspectives. EMJ Gastroenterol., 6, 90–97. doi: 10.33590/emjgastroenterol/10312003

[ref38] Zhangabylov А. Nurgalieva Z. Almukhanbetova R. (2002). “Results of serological studies to detect *Helicobacter pylori* infection in residents of Alma-Ata” in International gastroenterological congress, Almaty: International Gastroenterological Congress Proceedings, 66.

